# Generalized Forms of the Kraft Inequality for Finite-State Encoders

**DOI:** 10.3390/e28030278

**Published:** 2026-03-01

**Authors:** Neri Merhav

**Affiliations:** The Viterbi Faculty of Electrical and Computer Engineering, Technion–Israel Institute of Technology, Technion City, Haifa 3200003, Israel; merhav@ee.technion.ac.il; Tel.: +972-4-8294737

**Keywords:** source coding, finite-state machines, Kraft inequality, spectral radius, joint spectral radius

## Abstract

We derive a few extended versions of the Kraft inequality for information lossless finite-state encoders. The main basic contribution is in defining a notion of a Kraft matrix and in establishing the fact that a necessary condition for information losslessness of a finite-state encoder is that none of the eigenvalues of this matrix have modulus larger than unity, or equivalently, the spectral radius of the Kraft matrix cannot exceed one. We then derive several equivalent forms of this condition, which are based on well-known formulas for spectral radius. Even stronger results are presented for the important special case where the finite-state encoder is assumed irreducible. Finally, two extensions are outlined—one concerns the case of side information available to both encoder and decoder, and the other is for lossy compression.

## 1. Introduction

Kraft’s inequality plays a pivotal role in information theory. It provides a complete and elegant characterization of the feasibility of variable-length uniquely decodable (UD) codes by imposing a simple constraint on codeword lengths. In 1949, Kraft [1] introduced this inequality for prefix codes, establishing a condition on codeword lengths necessary for prefix decodability. Seven years later, McMillan [2] generalized this to UD codes, leading to the Kraft–McMillan inequality, which is widely used in information theory, first and foremost, to furnish a necessary and sufficient condition for the existence of a UD code with a given code-length function, and thereby also to prove the converse to the lossless source coding theorem, asserting that no UD source code can yield a coding rate below the entropy rate of the source. Once this necessary and sufficient condition is satisfied, there exists not only a general UD code, but also more specifically, a prefix code with that length function. Beyond its immediate operational meaning, Kraft’s inequality underlies many fundamental principles in lossless compression, such as the equivalence between lossless source coding and probability assignment. In general, its importance stems from the fact that it connects combinatorial properties of codes with analytical bounds in a precise and tractable manner. Classical treatments can be found in standard texts such as [3,4].

When memory is introduced into the encoder, however, the classical Kraft inequality (CKI) no longer applies directly. Finite-state (FS) encoders constitute a natural and widely studied model for compression with memory, arising in universal source coding, individual-sequence coding, and FS prediction. In this setting, the encoder’s output depends not only on the current source symbol, but also on an internal state that evolves over time in a manner that depends on past inputs. As a result, the set of admissible codeword length assignments is no longer characterized by a single scalar inequality, and the extension of Kraft’s condition becomes substantially more subtle.

Significant progress in this direction was made by Ziv and Lempel [5], who derived a generalized Kraft inequality (GKI) for information-lossless (IL) FS encoders by considering blocks over large super-alphabets, see Lemma 2 in [5]. When reading Ziv and Lempel’s article, the reader might get the impression that their GKI was established merely as an auxiliary result needed in their way of proving that the FS compressibility of a sequence is lower bounded by its asymptotic empirical entropy. Their focus was not on the Kraft inequality in its own right. Consequently, their formulation of Kraft’s inequality suffers from two main limitations: (i) it does not reduce exactly to the CKI when the encoder has merely one state, and (ii) it is based on super-alphabet extensions to long blocks rather than being formulated in a single-letter manner, or at the level in which the encoder is defined in the first place. More precisely, while the inequality remains valid even for short block lengths, it yields tight results only asymptotically for long blocks. But nevertheless, a direct, state-level generalization of Kraft’s inequality that mirrors the simplicity and sharpness of the classical result has remained elusive.

In this paper, we present several new forms of GKIs for IL FS encoders. Our approach associates with every given IL FS encoder a nonnegative matrix, termed the *Kraft matrix*, whose entries are determined by the encoder’s single-symbol output lengths and state transitions. We show that information losslessness imposes a spectral-radius constraint on this matrix, which serves as a natural analog of Kraft’s inequality. Unlike Ziv and Lempel’s GKI mentioned above, this inequality, as well as its several equivalent forms presented herein, reduces exactly to the CKI in the single-state case and avoids the use of super-alphabet extensions.

We then further refine the analysis for irreducible FS encoders, where the Perron–Frobenius theory yields stronger, uniform bounds on matrix powers. These results lead to transparent lower bounds on achievable compression rates for both stochastic sources and individual sequences. In addition, we extend the framework to settings with side information (SI) available at both the encoder and decoder, where the relevant constraint is expressed in terms of the joint spectral radius (JSR) of a finite set of Kraft matrices [6]. This extension clarifies the structural limitations imposed by SI and highlights the role of common sub-invariant vectors. Finally, another extension is associated with lossy source coding in the spirit of those of [7,8,9].

Overall, the proposed framework provides a unified and exact characterization of feasibility conditions for FS encoders, sharpening existing results and offering new tools for the analysis of compression and prediction under finite-memory constraints.

The outline of the remaining part of this article is as follows. In Section 2, we establish notation conventions, define the setting, and provide some background on the GKI of Ziv and Lempel. In Section 3, we present our basic GKI, asserting that the spectral radius of the Kraft matrix must not exceed unity for an IL FS encoder. Stronger and more explicit statements are then provided for irreducible encoders in Section 4. In Section 5, we apply the GKI of Section 4 to obtain converse bounds on compression and prediction of irreducible machines, both in the probabilistic setting and for individual sequences. Finally, in Section 6, we extend the GKI to the case of availability of SI, and in Section 7, we extend it to the lossy case.

## 2. Notation, Setting and Background

Throughout this paper, scalar random variables (RV’s) will be denoted by capital letters, their sample values will be denoted by the respective lower case letters, and their alphabets will be denoted by the respective calligraphic letters. A similar convention will apply to random vectors and their sample values, which will be denoted with the same symbols superscripted by the dimension. Thus, for example, $X^{n}$ (*n* – positive integer) will denote a random *n*-vector $\left(X_{1},\ldots ,X_{n}\right)$, and $x^{n}=\left(x_{1},\ldots ,x_{n}\right)$ is a specific vector value in $X^{n}$, the *n*–th Cartesian power of X, which is the alphabet of each component of $x^{n}$. For two positive integers, *i* and *j*, where i≤j, $x_{i}^{j}$ and $X_{i}^{j}$ will designate segments $\left(x_{i},\ldots ,x_{j}\right)$ and $\left(X_{i},\ldots ,X_{j}\right)$, respectively, where for i=1, the subscript will be omitted (as above). For i>j, $x_{i}^{j}$ (or $X_{i}^{j}$) will be understood as the null string. An infinite sequence $\left(x_{1},x_{2},\ldots \right)$ will be denoted by ***x***. Logarithms and exponents, throughout this paper, will be understood to be taken to the base 2 unless specified otherwise. The indicator function of an event A will be denoted by I{A}, i.e., I{A}=1 if A occurs and I{A}=0 if not.

Following the FS encoding model of [5], an FS encoder is defined by the quintuple, E=(X,Y,Z,f,g), whose five ingredients are defined as follows:

X is the finite alphabet of each symbol of the source sequence to be compressed. The cardinality of X will be denoted by α.
Y is a finite collection of binary variable-length strings, which is allowed to consist of empty string, denoted ‘null’ (whose length is zero);
Z is a finite set of *s* states of the encoder;
f:Z×X→Y is the output function, and
g:Z×X→Z is the next-state function.

Given an infinite source sequence to be compressed, $x=(x_{1},x_{2},\ldots )$, with $x_{i}\in X$, the FS encoder *E* produces an infinite output sequence, $y=(y_{1},y_{2},\ldots )$ with $y_{i}\in Y$, forming the compressed bit-stream, while passing through a sequence of states $z=(z_{1},z_{2},\ldots )$ with $z_{i}\in Z$, i=1,2,…. The encoder is governed by the recursive equations:

(1) $$\begin{array}{cccc} \; & y_{i} & = & f(z_{i},x_{i}), \end{array}$$

(2) $$\begin{array}{ccc} z_{i+1} & = & g(z_{i},x_{i}), \end{array}$$

for i=1,2,…, with a fixed initial state $z_{1}\in Z$. If at any step $y_{i}=null$, this is referred to as idling as no output is generated, but only the state evolves in response to the input. At each time instant *i*, the encoder emits $L\left(y_{i}\right)=L\left[f\left(z_{i},x_{i}\right)\right]$ bits, and it is understood that L(null)=0.

**Remark** **1.** *The null string option (which also appears in [5]) is motivated by the wish to allow the encoder to “idle” for certain combinations of inputs and states rather than “enforcing” it to output compressed bits at each and every time instant. This idling option opens the door to having a lot more flexibility and sometimes it is even necessary. For example, even when considering a simple block code as an example of an FS encoder (as will be done in the sequel in Example 1), then, in general, the encoder can output nothing before having read the entire input block. So formally, if the block length is k, then the encoder idles for k−1 time instants, and only upon reading the last input symbol, it produces the compressed codeword for that block.*

An encoder with *s* states, henceforth called an *s*-state encoder, is one for which |Z|=s. For the sake of simplicity, we adopt a few notation conventions from [5]: given a segment of input symbols $x_{i}^{j}$, where *i* and *j* are positive integers with i≤j, and an initial state $z_{i}$, we use $f(z_{i},x_{i}^{j})$ to denote the corresponding output segment $y_{i}^{j}$ produced by *E*. Similarly, $g(z_{i},x_{i}^{j})$ will denote the final state $z_{j+1}$ after processing the inputs $x_{i}^{j}$, beginning from state $z_{i}$. Thus, in response to an input $x^{n}$, the encoder produces a compressed bit string of length $L\left(y^{n}\right)=L\left[f\left(z_{1},x^{n}\right)\right]=\sum_{i=1}^{n}L\left[f\left(z_{i},x_{i}\right)\right]=\sum_{i=1}^{n}L\left(y_{i}\right)$ bits.

**Definition** **1.** *An FS encoder E is called IL if, given any initial state $z_{i}\in Z$, any positive integer n, and any input string, $x_{i}^{i+n}$, the triplet $\left(z_{i},f\left(z_{i},x_{i}^{i+n}\right),g\left(z_{i},x_{i}^{i+n}\right)\right)$ uniquely determines the corresponding input string $x_{i}^{i+n}$.*

**Remark** **2.** *The IL property can be considered as the FS counterpart of the notion of unique decodability for ordinary single-state codes (that is, codes with no memory). Indeed, every UD code can be viewed as a single-state IL encoder. But in general, an FS code is not necessarily prefix-free, because the codewords emitted at each time instant may depend on the internal state, which carries over additional information. It should be stressed that the IL property (required for each and every i and n) is attributed merely to the encoder, and it has nothing necessary to do with the mode of operation of the decoder. In particular, one may wonder why the final state, $g(z_{i},x_{i}^{i+n})$, plays a role in the ‘reconstruction’ of $x_{i}^{i+n}$ as defined in Definition 1. The final state is needed because an FS encoder can “carry information forward” in its state instead of emitting it out immediately. Consequently, without knowing the final state, some of the input information may still be stored in the encoder’s memory rather than in the emitted bits. It is easy to see this even in the above-mentioned simple example of a UD block code when viewed as an instance of an FS encoder: one can verify that the IL property holds in this case, and the reconstruction of the input according to Definition 1 indeed requires the final state in general (for details, the reader is referred to the discussion between Equations (15) and (16) in [10]).*

In Lemma 2 of [5], Ziv and Lempel presented a GKI for IL FS encoders. It asserts that for every IL encoder with *s* states and every positive integer *ℓ*,

(3) $$\sum_{x^{\ell }\in X^{\ell }}2^{-\min_{z\in Z}L\left[f\left(z,x^{\ell }\right)\right]}\leq s^{2}\left[1+\log\left(1+\frac{\alpha^{\ell }}{s^{2}}\right)\right],$$

where we remind the reader that α is the alphabet size of the input sequence to be compressed. Ziv and Lempel’s GKI was a perfect tool for their purpose of proving that the compression ratio achieved by an IL FS encoder cannot be smaller than the asymptotic empirical entropy rate (defined in [5]) for any infinite source sequence ***x***. However, when examined for finitely long sequences, and from the perspective of serving as a necessary condition for information losslessness, this inequality suffers from two main weaknesses.

1. It does not exactly recover the CKI for the special case, s=1, as in that case, the right-hand side (r.h.s.) becomes $1+\log(1+\alpha^{\ell })>1$. Moreover, even if ℓ=1, the right-hand side (r.h.s.), which is $1+\log_{2}\left(1+\alpha \right)$, is even larger than 2 for every α≥2. On a related note, a close inspection of the proof of Lemma 2 in [5] reveals that the inequality in Equation (3) is actually a strong inequality (<), in other words, this inequality is always loose.
2. It is significant only upon an extension from single symbols into the super-alphabet of *ℓ*-strings for large *ℓ*, unlike the ordinary Kraft inequality, which is asserted in the same level that the code is defined. For example, the CKI for a code that is defined in the level of single symbols of X is asserted in that level, i.e., $\sum_{x\in X}2^{-L[f(x)]}\leq 1$.

Our objective in this work is first and foremost to establish another GKI for IL FS encoders that is free of the above-mentioned drawbacks. In other words, for the case s=1, it would recover the traditional Kraft inequality exactly, and it will be posed in the single-letter level without recourse to alphabet extensions. The latter property will enable one to verify relatively easily that this inequality holds in a given situation.

Our first proposed GKI serves as the basis for our subsequent derivations. Having derived it, we then confine attention to the subclass of irreducible IL FS encoders, namely, FS encoders for which every state can be reached from every state in a finite number of steps. For this important subclass of encoders, we provide several alternative formulations of the GKI and provide a stronger upper bound to the growth rate of the Kraft sum as function of the block length. Again, all these forms are smooth extensions of the CKI in the sense that in the special case s=1, they degenerate to the CKI. Finally, we consider extensions in two directions (one at a time): the first is the case where SI is available to both encoder and decoder, and the second is the case of lossy compression.

## 3. The Basic Generalized Kraft Inequality

For a given IL FS encoder *E* with *s* states, let us define an s×s Kraft matrix *K*, whose $\left(z,z^{\prime }\right)$ entry is given by

(4) $$K_{zz^{\prime }}=\sum_{\left\{x:\;g\left(z,x\right)=z^{\prime }\right\}}2^{-L[f(z,x)]},\;\;\;\;\;\left(z,z^{\prime }\right)\in Z^{2},$$

where the summation over an empty set is understood as zero. Since *K* is a non-negative matrix, then according to Theorem 8.3.1 in [11], the spectral radius of *K*, ρ(K), is an eigenvalue of *K*. (We remind the reader that the spectral radius is the maximum absolute value (magnitude) of the eigenvalues of a square matrix.)

Our first form of a GKI is the following.

**Theorem** **1.** *For every IL FS encoder,*

(5) ρ(K)≤1.

As can be seen, this GKI has the two desired properties we mentioned above:

1. The case s=1 obviously recovers the CKI, since in this case, *K* degenerates to a scalar, which is nothing but the Kraft sum, $\sum_{x\in X}2^{-L[f(x)]}$, and then Equation (5) asserts that $\sum_{x\in X}2^{-L[f(x)]}\leq 1$, as desired.
2. The matrix *K* is defined in terms of the functions *f* and *g* only. These functions are defined in the level of the single symbols and states.

The first property sets the stage of establishing the condition ρ(K)≤1 as a *necessary condition for information losslessness* of a given FS encoder, in analogy to the fact that ordinary Kraft inequality is a necessary (and sufficient) condition for the existence of unique decodability in the case s=1. Since there is no involvement of summations over super-alphabets of long vectors, this condition is relatively easy to check, similarly as the CKI, which is a necessary condition for the unique decodability property of ordinary lossless source codes.

**Proof.**  The proof is in the footsteps of Karush [12]. Let $L_{\max}\overset{▵}{=}\max_{z,x}L\left[f\left(z,x\right)\right]$. For every positive integer *ℓ*, the $\left(z,z^{\prime }\right)$ entry of the *ℓ*-th order power, $K^{\ell }$, is given by

(6) $$\begin{array}{ccc} {\left[K^{\ell }\right]}_{zz^{\prime }} & = & \sum_{z_{2}\in Z}\sum_{z_{3}\in Z}\cdots \sum_{z_{\ell }\in Z}\prod_{i=1}^{\ell }\left(\sum_{\left\{x_{i}:\;g\left(z_{i},x_{i}\right)=z_{i+1}\right\}}2^{-L[f\left(z_{i},x_{i}\right)]}\right)\\ & = & \sum_{\left\{x^{\ell }:\;g\left(z,x^{\ell }\right)=z^{\prime }\right\}}2^{-L[f\left(z,x^{\ell }\right)]}\\ & = & \sum_{l=0}^{\ell \cdot L_{\max}}2^{-l}\cdot \left|\left\{x^{\ell }:\;L\left[f\left(z,x^{\ell }\right)\right]=l,\;g\left(z,x^{\ell }\right)=z^{\prime }\right\}\right|\\ & \leq & \sum_{l=0}^{\ell \cdot L_{\max}}2^{-l}\cdot 2^{l}\\ & = & 1+\ell \cdot L_{\max}, \end{array}$$

where in the first line, $z_{1}=z$ and $z_{\ell +1}=z^{\prime }$, and the inequality is due to the postulated IL property (as *z* and $z^{\prime }$ are fixed). Alternatively, we can also bound ${\left[K^{\ell }\right]}_{zz^{\prime }}$ by $1+\log(1+\alpha^{\ell })$ using the same considerations as in the proof of Lemma 2 in [5], except that the factor $s^{2}$ is missing since *z* and $z^{\prime }$ are fixed. The choice of which is better between these two bounds depends, of course, on $L_{\max}$. In any case, both expressions are essentially linear in *ℓ*. Continuing with the first bound, it follows that

(7) $$\sum_{z^{\prime }\in S}{\left[K^{\ell }\right]}_{zz^{\prime }}=\sum_{x^{\ell }\in X^{\ell }}2^{-L[f\left(z,x^{\ell }\right)]}\leq s\left(1+\ell \cdot L_{\max}\right).$$

Let $e_{z}$ be a column vector of dimension *s* whose entries are all zero except the entry corresponding to state *z*, which is 1, and let **1** denote the all-one column vector of dimension *s*. Then, Equation (7) can be rewritten as

(8) $$e_{z}^{⊤}K^{\ell }1\leq s\left(1+\ell \cdot L_{\max}\right).$$

To prove that ρ(K)≤1, we proceed by contradiction. Assume conversely, that $\lambda \overset{▵}{=}\rho \left(K\right)>1$. Since *K* has non-negative entries, the Perron–Frobenius theorem (see again Theorem 8.3.1 in [11]) guarantees that the right eigenvector *v* corresponding to λ has non-negative components and at least one strictly positive component. Since $1={\left(1,\ldots ,1\right)}^{T}$ has strictly positive components, there exists a constant δ>0 such that 1≥δv component-wise. Multiplying by $K^{\ell }$ from the left and using the non-negativity of *K*, we obtain

(9) $$K^{\ell }1\geq \delta K^{\ell }v=\delta \lambda^{\ell }v.$$

Taking the *z*-th component yields

(10) $$e_{z}^{⊤}K^{\ell }1\geq \delta \lambda^{\ell }v_{z}.$$

For any index *z* with $v_{z}>0$, the r.h.s. grows exponentially in *ℓ* since λ>1, but this contradicts Equation (8) which establishes an upper bound that grows only linearly in *ℓ*. Therefore the postulate ρ(K)>1 cannot hold true, and we conclude that ρ(K)≤1, which completes the proof. □

Since ρ(K)≤1, it is clear that for every natural *ℓ*, $\rho \left(K^{\ell }\right)={\left[\rho \left(K\right)\right]}^{\ell }\leq \rho \left(K\right)\leq 1$. In other words, the spectral radius of

(11) $$K^{\ell }={\left\{\sum_{\left\{x^{\ell }:\;g\left(z,x^{\ell }\right)=z^{\prime }\right\}}2^{-L[f\left(z,x^{\ell }\right)]}\right\}}_{z,z^{\prime }\in Z}$$

is also never larger than unity, which is an extension of our GKI to super-alphabets, which is again, a smooth extension that degenerates to the CKI for s=1.

**Figure 1:**
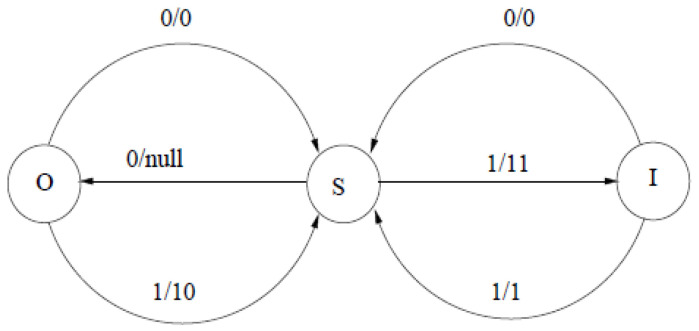
State transition diagram of the encoder in Example 1. The various state transitions are labeled in a form x/y, where *x* denotes the input and y=f(z,x) denotes the output.

**Example** **1.** *Consider a binary source sequence and a block code of length 2, which maps the source strings 00, 01, 10, and 11, into 0, 10, 110, and 111, respectively. This code can be implemented by an FS encoder with s=3 states, labeled ‘S’, ‘O’, and ‘I’, using the following functions, f and g (see also Figure 1):*

$$\begin{array}{ccc} g(S,0) & = & O,\\ g(S,1) & = & I,\\ g(O,0) & = & g(O,1)=g(I,0)=g(I,1)=S, \end{array}$$

*and*

$$\begin{array}{ccc} f(S,0) & = & null,\\ f(S,1) & = & 11,\\ f(O,0) & = & 0,\\ f(O,1) & = & 10,\\ f(I,0) & = & 0,\\ f(I,1) & = & 1. \end{array}$$

*State ‘S’ designates the start of a block. State ‘O’ remembers that the first input of the block was ‘0’ and state ‘I’ remembers that the first input was ‘1’. Upon moving to state ‘I’, the encoder can already output ‘11’, because the entire codeword will be either ‘110’ or ‘111’ if the first source symbol is ‘1’, so the first two coded bits are ‘11’ in either case. After state ‘I’, the encoder can complete the codeword according to the second input in the block. After state ‘O’, outputs are generated only upon receiving the second symbol. After both states ‘O’ and ‘I’, the encoder must return to state ‘S’ in order to start the next block. The corresponding Kraft matrix (with row and column indexing in the order of (S,O,I)) is given by:*

(12) $$K=\left(\begin{array}{ccc} 0 & 2^{-0} & 2^{-2}\\ 2^{-1}+2^{-2} & 0 & 0\\ 2^{-1}+2^{-1} & 0 & 0 \end{array}\right)=\left(\begin{array}{ccc} 0 & 1 & 0.25\\ 0.75 & 0 & 0\\ 1 & 0 & 0 \end{array}\right)$$

*whose eigenvalues are 1, 0, and −1, and so the spectral radius is ρ(K)=1. As can be seen, the sums of the second and third rows do not exceed unity, so when the initial state is either ‘O’ or ‘I’, the Kraft sum does not exceed 1. On the other hand, the Kraft sum corresponding to the first row (pertaining to ‘S’) exceeds unity. This demonstrates an important observation: the model of a general IL FS encoder is broader and more general than a model of an FS encoder for which given every state, the encoder implements a certain prefix (or UD) code for the variety of incoming symbols. For ℓ=100, we find that*

$$K^{100}=\left(\begin{array}{ccc} 1 & 0 & 0\\ 0 & 0.75 & 0.1875\\ 0 & 1 & 0.25 \end{array}\right)$$

*where eigenvalues are 0, 1, and 1. Here, the sums of the first and the second rows do not exceed unity, so when the initial state is either ‘S’ or ‘O’, the Kraft sum does not exceed 1. On the other hand, the Kraft sum corresponding to the third row exceeds unity, and so, the above comment with regard to K applies here too. This concludes Example 1.*

Earlier, we said that ρ(K)≤1 is a necessary condition for a given code with next-state function *g* and code-lengths {L[f(z,x)]} to be IL. One might naturally wonder whether it is also a sufficient condition. This question is open in general, but we have two comments related to this issue.

The first is that the answer is obviously affirmative for the subclass of IL encoders, which satisfies the CKI for each and every state, i.e., $\sum_{z^{\prime }\in Z}K_{zz^{\prime }}=\sum_{x\in X}2^{-L[f(z,x)]}\leq 1$: Simply construct a separate prefix code with length function {L[f(z,x)],x∈X} for each z∈Z. However, in general, an IL code does not necessarily satisfy the ordinary Kraft inequality for each *z*. Indeed, in Example 1, the sum of the first row of *K* is larger than 1.

The second comment is that we can give an affirmative answer in the level of longer blocks. Let $z_{1}\in Z$ be an arbitrary initial state and consider the lengths, $l\left(z_{1},x^{n}\right)=\sum_{i=1}^{n}L\left[f\left(z_{i},x_{i}\right)\right]$. Then, as we have seen in (6):

(13) $$\sum_{x^{n}}2^{-L[f\left(z_{1},x^{n}\right)]}\leq s\left(1+nL_{\max}\right),$$

where the factor of *s* stems from taking the sum of $K_{zz^{\prime }}$ over $z^{\prime }\in Z$. Equivalently,

(14) $$\sum_{x^{n}}2^{-[l\left[f\left(z_{1},x^{n}\right)\right]+\log\left[s\left(1+nL_{\max}\right)\right]}\leq 1,$$

and so, there exists a prefix code with lengths $l^{\prime }\left(x^{n}\right)=l\left(z_{1},x^{n}\right)+\log\left[s\left(1+nL_{\max}\right)\right]+\log s$, which are relatively only slightly longer than those of the original code. Here, the additional logs term is a header that notifies $z_{1}$.

## 4. Irreducible FS Encoders

IL FS encoders for which the next-state function *g* allows transition from every state to every state within a finite number of steps, are henceforth referred to as *irreducible FS encoders*. Equivalently, defining the s×s adjacency matrix *A* such that $A_{zz^{\prime }}=1$ whenever ∃x∈X such that $g\left(z,x\right)=z^{\prime }$ and $A_{zz^{\prime }}=0$ otherwise, then an IL FS encoder is irreducible if the matrix *A* is irreducible. Likewise, an IL FS encoder is irreducible if the matrix *K* is irreducible. For an irreducible FS encoder, the shortest path from every state *z* to every other state $z^{\prime }$ lasts no longer than s−1 steps, because any longer path must visit a certain state $z^{\prime \prime }$ at least twice, meaning that this path contains a loop starting and ending at $z^{\prime \prime }$, which can be eliminated. Clearly, the encoder of Example 1 is irreducible.

Intuitively, it makes sense to use irreducible encoders, because for reducible ones, once the machine leaves a certain subset of transient states, it can never return, and so, effectively, reducible encoders use eventually a smaller number of states after finite time. Specifically, given a reducible machine and an infinite individual sequence $x_{1},x_{2},\ldots$, suppose the machine starts at a transient state. Then, there are two possibilities: either the machine quits the subset of transient states after finite time, or it stays in that subset forever. In the former case, the transient states are in use for finite time only and then never used again. In the latter case, the recurrent states are never used. In either case, asymptotically, only a subset of the available states are used, and so, effectively, the number of states actually used is smaller than *s*. Let $Z_{\infty }$ denote the set of states visited infinitely many times along the sequence. This set is necessarily closed and induces a strongly connected subgraph. Consequently, the asymptotic behavior of the encoder along the given sequence is governed entirely by its restriction to $Z_{\infty }$, which constitutes an irreducible FS encoder with strictly fewer than *s* states. Therefore, reducible encoders cannot offer asymptotic advantages over irreducible ones, even for individual sequences.

Assume next that the next-state function *g* induces an irreducible matrix $K^{\ell }$, where *ℓ* be an arbitrary positive integer. Since $K^{\ell }$ is non-negative and irreducible, the Collatz–Wielandt formulas [13,14] for the spectral radius of $K^{\ell }$ hold true. These are given by

(15) $$\rho \left(K^{\ell }\right)=\max_{w\in W_{+}}\min_{\left\{z:\;w_{z}>0\right\}}\frac{{\left[K^{\ell }w\right]}_{z}}{w_{z}}=\min_{w\in W_{+}}\max_{\left\{z:\;w_{z}>0\right\}}\frac{{\left[K^{\ell }w\right]}_{z}}{w_{z}}.$$

where *w* is an *s*-dimensional column vector and $W_{+}$ is the set of all such vectors with non-negative components not all of which are zero. These lead to the two following GKIs:

(16) $$\forall \;w\in W_{+}\;\exists \;z\;\mathrm{such}\;\mathrm{that}\;w_{z}>0\;\mathrm{and}\;\sum_{z^{\prime }\in S}w_{z^{\prime }}\cdot \sum_{\left\{x^{\ell }:\;g\left(z,x^{\ell }\right)=z^{\prime }\right\}}2^{-L[f\left(z,x^{\ell }\right)]}\leq w_{z},$$

and

(17) $$\exists \;w\in W_{+}\;\forall \;z\;\mathrm{such}\;\mathrm{that}\;w_{z}>0:\;\sum_{z^{\prime }\in S}w_{z^{\prime }}\cdot \sum_{\left\{x^{\ell }:\;g\left(z,x^{\ell }\right)=z^{\prime }\right\}}2^{-L[f\left(z,x^{\ell }\right)]}\leq w_{z},$$

The first formulation can be simplified at the price of a possible loss of tightness, by selecting *w* to be the all-one vector and thereby bounding $\rho (K^{\ell })$ from below. This results in the conclusion that an IL FS encoder always satisfies yet another GKI:

(18) $$\exists z\in S\;\sum_{x^{\ell }\in X^{\ell }}2^{-L[f\left(z,x^{\ell }\right)]}\leq 1.$$

In words, for every given irreducible FS encoder, (f,g), and for every natural *ℓ*, there is at least one initial state, z∈Z, for which the Kraft sum is less than unity, but again, not all states must satisfy this condition (as we saw in Example 1, the Kraft sum exceeds unity when the initial state is ‘S’). All these are also smooth extensions of the CKI in the sense that for s=1 we are back to the CKI.

But there is an even stronger GKI that applies to irreducible encoders. It asserts that in the irreducible case, $K^{n}$ does not even grow linearly as in (6), but is rather bounded by a constant, independent of *n*. For s=1, this constant is 1, again in agreement with the CKI.

**Theorem** **2.** *Let K be an irreducible Kraft matrix. Then, for all $z,z^{\prime }\in Z$ and for every natural n,*

(19) $${\left(K^{n}\right)}_{zz^{\prime }}=\sum_{\left\{x^{n}:\;g\left(z,x^{n}\right)=z^{\prime }\right\}}2^{-L[f\left(z,x^{n}\right)]}\leq 2^{\left(s-1\right)L_{\max}}.$$

*Consequently, for every z∈S,*

(20) $$\sum_{x^{n}\in X^{n}}2^{-L[f\left(z,x^{n}\right)]}\leq s\cdot 2^{\left(s-1\right)L_{\max}},$$

*and*

(21) $$\sum_{z\in S}\sum_{x^{n}\in X^{n}}2^{-L[f\left(z,x^{n}\right)]}\leq s^{2}\cdot 2^{\left(s-1\right)L_{\max}}.$$

**Proof.**  It is sufficient to prove the first inequality, as the two other ones will follow trivially by a summation over $z^{\prime }\in Z$ and then also over z∈Z, respectively. Since *K* is non-negative and irreducible, the Perron–Frobenius theorem applies. This theorem asserts that the spectral radius, ρ(K), is positive and simple, with left and right eigenvectors, *u* and *v*, respectively, that have only strictly positive components. In Theorem 1 we have already proved that ρ(K)≤1. Assume first that ρ(K)=1. Then, $u^{T}K^{n}=u^{T}$, or, equivalently,

(22) $$\sum_{z\in S}u_{z}{\left(K^{n}\right)}_{zz^{\prime }}=u_{z^{\prime }}\;\;\;\;\forall \;z^{\prime }\in Z.$$

Since all terms are non-negative, the left-hand side is lower bound by $u_{z}{\left(K^{n}\right)}_{zz^{\prime }}$ for any z∈Z. This implies for every $z,z^{\prime }\in Z$

(23) $${\left(K^{n}\right)}_{zz^{\prime }}\leq \frac{u_{z^{\prime }}}{u_{z}}\leq \frac{\max_{z\in Z}u_{z}}{\min_{z\in Z}u_{z}}.$$

Let $z_{⋆}\in Z$ and $z^{⋆}\in Z$ be achievers of $\min_{z\in Z}u_{z}$ and $\max_{z\in Z}u_{z}$, respectively. Then, for every $z,z^{\prime }\in Z$,

(24) $${\left(K^{n}\right)}_{zz^{\prime }}\leq \frac{u_{z^{⋆}}}{u_{z_{⋆}}}.$$

Since *K* is irreducible and since $z^{⋆}$ and $z_{⋆}$ are distinct, there exists a path of length ℓ≤s−1 from $z^{⋆}$ to $z_{⋆}$, say, $z^{⋆}\to z_{1}\to \cdots \to z_{\ell -1}\to z_{⋆}$ such that

(25) $${\left(K^{\ell }\right)}_{z^{⋆}z_{⋆}}\geq K_{z^{⋆}z_{1}}\cdot K_{z_{1}z_{2}}\cdots K_{z_{\ell -1}z_{⋆}}>0.$$

Since all positive entries of *K* are at least as large as $2^{-L_{\max}}$, this product is at least as large as $2^{-\ell L_{\max}}\geq 2^{-\left(s-1\right)L_{\max}}$. It follows then that

(26) $${\left(K^{\ell }\right)}_{z^{⋆}z_{⋆}}\geq 2^{-\left(s-1\right)L_{\max}}.$$

Now,

(27) $$u_{z_{⋆}}=\sum_{z\in S}u_{z}{\left(K^{\ell }\right)}_{zz_{⋆}}\geq u_{z^{⋆}}{\left(K^{\ell }\right)}_{z^{⋆}z_{⋆}}\geq u_{z^{⋆}}2^{-\left(s-1\right)L_{\max}},$$

which implies that

(28) $$2^{\left(s-1\right)L_{\max}}\geq \frac{u_{z^{⋆}}}{u_{z_{⋆}}}\geq {\left(K^{n}\right)}_{zz^{\prime }},$$

for every $z,z^{\prime }\in S$. This completes the proof for the case ρ(K)=1. The case ρ(K)<1 is obtained from the case ρ(K)=1 by simply defining $\hat{K}=K/\rho \left(K\right)$ and using the fact that all non-negative entries of $\hat{K}$ are lower bounded by $2^{-L_{\max}}/\rho \left(K\right)$. Since $\hat{K}$ is also irreducible and since $\rho (\hat{K})=1$, we now have

(29) $${\left({\hat{K}}^{n}\right)}_{zz^{\prime }}\leq {\left[\rho \left(K\right)2^{L_{\max}}\right]}^{s-1}.$$

But ${\hat{K}}^{n}=K^{n}/{\left[\rho \left(K\right)\right]}^{n}$, and so,

(30) $${\left(K^{n}\right)}_{zz^{\prime }}\leq {\left[\rho \left(K\right)\right]}^{n+s-1}\cdot 2^{\left(s-1\right)L_{\max}}<2^{\left(s-1\right)L_{\max}}.$$

This completes the proof of Theorem 2. □

## 5. Converse Bounds Derived from the GKI

In this section, we demonstrate how the GKI of Section 4 can be used to obtain lower bounds on the performance of irreducible machines in compression and in prediction problems. For compression, both probabilistic sources and individual sequences are considered. For prediction, only the individual sequence version is presented, but the probabilistic counterpart can also be derived straightforwardly using the same ideas.

### 5.1. Compression of Probabilistic Sources

Let $\left\{P\left(z,x^{\ell }\right),\;z\in Z,\;x^{\ell }\in X^{\ell }\right\}$ be a joint probability distribution of random variables *Z* and $X^{\ell }$. Then,

(31) $$\begin{array}{ccc} s^{2}\cdot 2^{\left(s-1\right)L_{\max}} & \geq & \sum_{z\in Z}\sum_{x^{\ell }\in X^{\ell }}2^{-L[f\left(z,x^{\ell }\right)]}\\ & = & \sum_{z\in Z}\sum_{x^{\ell }\in X^{\ell }}P\left(z,x^{\ell }\right)\cdot 2^{-L\left[f\left(z,x^{\ell }\right)\right]-\log P\left(z,x^{\ell }\right)}\\ & \geq & \exp_{2}\left\{-\sum_{z\in Z}\sum_{x^{\ell }\in X^{\ell }}P\left(z,x^{\ell }\right)L\left[f\left(z,x^{\ell }\right)\right]+H\left(Z,X^{\ell }\right)\right\}\\ & = & \exp_{2}\left[-E\left\{L\left[f\left(Z,X^{\ell }\right)\right]\right\}+H\left(Z,X^{\ell }\right)\right], \end{array}$$

where the inequality follows from Jensen’s inequality and the convexity of the exponential function. By taking logarithms of both sides, rearranging terms, and normalizing by *ℓ*, we get

(32) $$\begin{array}{ccc} R & = & \frac{E\{L\left[f\left(Z,X^{\ell }\right)\right]\}}{\ell }\\ & \geq & \frac{H(Z,X^{\ell })}{\ell }-\frac{\log_{2}\left(s^{2}\cdot 2^{\left(s-1\right)L_{\max}}\right)}{\ell }\\ & \geq & \frac{H(X^{\ell })}{\ell }-\frac{2\log_{2}s+\left(s-1\right)L_{\max}}{\ell }, \end{array}$$

and if the source *P* is stationary, $H(X^{\ell })/\ell$ can be further lower bounded by $H(X_{\ell }|X^{\ell -1})$, to obtain

(33) $$R\geq H\left(X_{\ell }|X^{\ell -1}\right)-\frac{2\log_{2}s+\left(s-1\right)L_{\max}}{\ell }.$$

Since this bound applies to every positive integer *ℓ*, we may maximize the lower bound over *ℓ*, and obtain

(34) $$R\geq \underset{\ell \geq 1}{\sup}\left\{H\left(X_{\ell }|X^{\ell -1}\right)-\frac{2\log_{2}s+\left(s-1\right)L_{\max}}{\ell }\right\}.$$

We see that thanks to Theorem 2, the vanishing term subtracted from the entropy decays at the rate of 1/ℓ as opposed to the (logℓ)/ℓ rate that stems from Lemma 2 of [5] as well as from the more general inequality of $1+\ell \cdot L_{\max}$, that is obtained when reducible machines are allowed.

### 5.2. Compression of Individual Sequences

In the context of individual sequences, we can arrive at an analogous lower bound, provided that we define a shift-invariant empirical distribution. Specifically, let $x^{n}$ be a given individual sequence, let *ℓ* be a positive integer smaller than *n*, and let $z_{1}$ be a given initial state of the encoder. We assume that $x^{n}$ cyclic with respect to (w.r.t.) *g* in the sense that $g\left(z_{n},x_{n}\right)=z_{1}$. If this is not the case, consider an extension of $x^{n}$ by concatenating a suffix $x_{n+1}^{n+m}$ such that the extended sequence would be cyclic w.r.t. *g*. Since *g* is assumed irreducible, this is always possible and the length *m* of the extension need not be larger than s−1. To avoid cumbersome notation, we redefine $x^{n}$ to be the sequence after the cyclic extension (if needed), and we shall keep in mind that this cyclic extension adds no more than $m\cdot L_{\max}\leq \left(s-1\right)L_{\max}$ bits to the compressed description, or equivalently, $\left(s-1\right)L_{\max}/n$ to the compression ratio, and so, this extra rate should be subtracted back upon returning to the original sequence before the cyclic extension. For every $w^{\ell }\in X^{\ell }$ and z∈S, let

(35) $$\delta \left(z_{i},x_{i}^{((i-1)⊕(\ell -1))+1};z,w^{\ell }\right)=\left\{\begin{array}{cc} 1 & z_{i}=z\;\mathrm{and}\;x_{i}^{((i-1)⊕(\ell -1))+1}=w^{\ell }\\ 0 & \mathrm{elsewhere} \end{array}\right.$$

where ⊕ denotes modulo-*n* addition. Next, define the empirical distribution

(36) $$\hat{P}\left(z,w^{\ell }\right)=\frac{1}{n}\sum_{i=1}^{n}\delta \left(z_{i},x_{i}^{((i-1)⊕(\ell -1))+1};z,w^{\ell }\right),$$

Now,

(37) $$\begin{array}{ccc} \frac{1}{n}\sum_{i=1}^{n}L\left[f\left(z_{i},x_{i}\right)\right] & = & \frac{1}{n\ell }\sum_{i=1}^{n}\ell \cdot L\left[f\left(z_{i},x_{i}\right)\right]\\ & = & \frac{1}{n\ell }\sum_{i=1}^{n}\sum_{j=0}^{\ell -1}L\left[f\left(z_{i},x_{((i-1)⊕j)+1}\right)\right]\\ & = & \frac{1}{n\ell }\sum_{i=1}^{n}L\left[f\left(z_{i},x_{i}^{((i-1)⊕(\ell -1))+1}\right)\right]\\ & = & \frac{1}{n\ell }\sum_{i=1}^{n}\sum_{z\in S}\sum_{w^{\ell }\in X^{\ell }}\delta \left(z_{i},x_{i}^{((i-1)⊕(\ell -1))+1};z,w^{\ell }\right)L\left[f\left(z,w^{\ell }\right)\right]\\ & = & \frac{1}{n\ell }\sum_{z\in S}\sum_{w^{\ell }\in X^{\ell }}\sum_{i=1}^{n}\delta \left(z_{i},x_{i}^{((i-1)⊕(\ell -1))+1};z,w^{\ell }\right)L\left[f\left(z,w^{\ell }\right)\right]\\ & = & \frac{1}{\ell }\sum_{z\in S}\sum_{w^{\ell }\in X^{\ell }}\hat{P}\left(z,w^{\ell }\right)L\left[f\left(z,w^{\ell }\right)\right]\\ & \geq & \hat{H}\left(X_{\ell }|X^{\ell -1}\right)-\frac{2\log_{2}s+\left(s-1\right)L_{\max}}{\ell }, \end{array}$$

where $\hat{H}\left(X_{\ell }|X^{\ell -1}\right)$ is the empirical conditional entropy derived from the shift-invariant distribution $\hat{P}$. The last inequality follows from a similar derivation as in the probabilistic case considered above, except that the earlier distribution *P* is now replaced by the empirical one, $\hat{P}$, and therefore the corresponding entropies are replaced by their empirical counterparts. Using the fact that this is true for every natural ℓ<n and returning to the original sequence before the cyclic extension, we find that

(38) $$\frac{1}{n}\sum_{i=1}^{n}L\left[f\left(z_{i},x_{i}\right)\right]\geq \max_{1\leq \ell <n}\left\{\hat{H}\left(X_{\ell }|X^{\ell -1}\right)-\frac{2\log s+\left(s-1\right)L_{\max}}{\ell }\right\}-\frac{\left(s-1\right)L_{\max}}{n}.$$

Furthermore, invoking Ziv’s inequality (see Equation (13.125) in [4]), this can be further lower bounded in terms of the LZ complexity. Specifically, according to Equation (13.125) in [4], for every Markov source, $Q_{\ell -1}$, of order ℓ−1 and every $x^{n}\in X^{n}$,

(39) $$c\left(x^{n}\right)\log c\left(x^{n}\right)\leq -\log Q_{\ell -1}\left(x^{n}|x_{-(\ell -2)}^{0}\right)+\epsilon_{\ell }\left(n\right),$$

where $c(x^{n})$ is the maximum number of distinct phrases whose concatenation forms $x^{n}$, and where $\epsilon_{\ell }\left(n\right)$ tends to zero at the rate of O(log(logn)/logn) for every fixed *ℓ*. By minimizing the r.h.s. w.r.t. $Q_{\ell -1}$, we get

(40) $$c\left(x^{n}\right)\log c\left(x^{n}\right)\leq n\hat{H}\left(X_{\ell }|X_{0}^{\ell -1}\right)+n\cdot \epsilon_{\ell }\left(n\right),$$

and so,

(41) $$\frac{1}{n}\sum_{i=1}^{n}L\left[f\left(z_{i},x_{i}\right)\right]\geq \frac{c\left(x^{n}\right)\log c\left(x^{n}\right)}{n}-\min_{\ell }\left[\epsilon_{\ell }\left(n\right)+\frac{2\log s+\left(s-1\right)L_{\max}}{\ell }\right]-\frac{\left(s-1\right)L_{\max}}{n}.$$

The minimizing *ℓ* can be found to be proportional to $\sqrt{n}$, but the dominant term of $\epsilon_{\ell }\left(n\right)$ remains of the order of $\frac{\log(\log n)}{\log n}$.

### 5.3. Prediction of Individual Sequences

**Figure 2:**
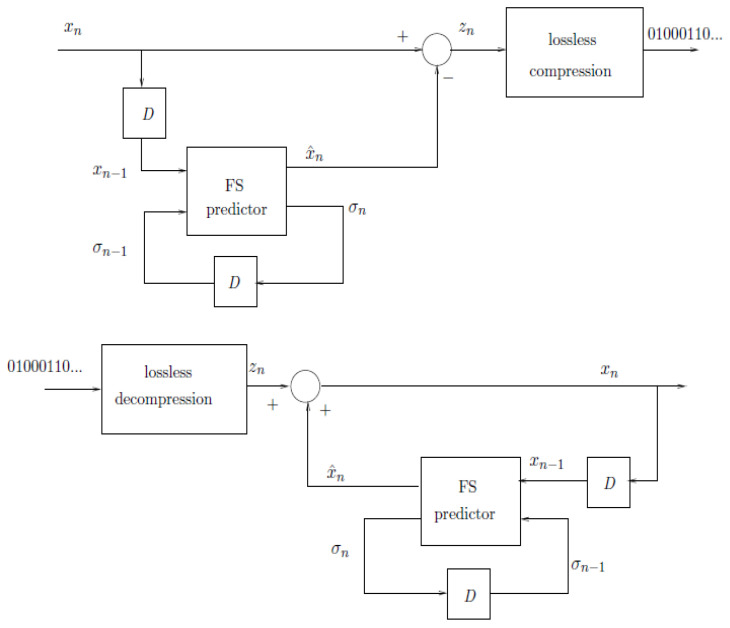
Auxiliary predictive encoder and decoder. The upper block diagram depicts the encoder that losslessly compresses the prediction error signal, $z_{n}$, which is the difference between the input signal, $x_{n}$, and its prediction, ${\hat{x}}_{n}$ obtained using an FS predictor. The lower block diagram stands for the corresponding decoder.

We next derive a lower bound to the prediction error of any FS predictor that is based on an irreducible FS machine. The idea is to harvest the compression lower bound to induce a lower bound on prediction by considering an FS encoder that is based on FS prediction and encoding the prediction error (predictive coding)—see Figure 2.

Consider an FS predictor with *q* states, defined by the following recursion, for i=1,2,…

(42) $$\begin{array}{ccc} {\hat{x}}_{i+1} & = & u(x_{i},\sigma_{i}),\\ \sigma_{i+1} & = & v(x_{i},\sigma_{i}), \end{array}$$

where $\sigma =(\sigma_{1},\sigma_{2},\ldots )$, $\sigma_{i}\in \Sigma$, i=1,2,…, is a corresponding infinite state sequence, whose alphabet, Σ, is a finite set of states of cardinality *q*, and $\hat{x}=\left({\hat{x}}_{1},{\hat{x}}_{2},\ldots \right)$, ${\hat{x}}_{i}\in X$, i=1,2,…, is the resulting predictor output sequence. Without loss of generality, the initial state, $\sigma_{1}$, and the initial prediction, ${\hat{x}}_{1}$, are assumed fixed members, $\sigma_{⋆}\in \Sigma$ and ${\hat{x}}_{⋆}\in X$, respectively. Here, u:X×Σ→X is the predictor output function and v:X×Σ→Σ is the next-state function.

It is assumed that X is a group with well-defined addition and subtraction operations. For example, if X={0,1,…,α−1} then it is natural to equip X with addition and subtraction modulo α. Let $\rho :X\to R^{+}$ denote a given loss function. Then, the performance of a predictor across the time range, 1≤t≤n is measured in terms of the time-average,

(43) $$\frac{1}{n}\sum_{i=1}^{n}\rho \left(x_{i}-{\hat{x}}_{i}\right).$$

Given an arbitrary irreducible FS predictor (u,v) as defined above, consider the auxiliary conditional probability distribution,

(44) $$Q_{\theta }\left(x_{i+1}|x_{i},\sigma_{i}\right)=\frac{e^{-\rho (x_{i+1}-u\left(x_{i},\sigma_{i}\right))/\theta }}{Z(\theta )},\;\;\theta \geq 0,$$

where

(45) $$Z\left(\theta \right)=\sum_{x\in X}e^{-\rho (x)/\theta }.$$

Define also the function

(46) $$\Delta \left(R\right)=\underset{\theta \geq 0}{\sup}\theta \cdot \left[R-\log Z\left(\theta \right)\right],\;\;\;R\geq 0.$$

Now, define

(47) $$Q_{\theta }\left(x^{n}\right)=\prod_{i=1}^{n}Q_{\theta }\left(x_{i}|x_{i-1},\sigma_{i-1}\right)$$

where $\sigma_{0}$ and $x_{0}$ are arbitrary members of Σ and X, respectively, such that $\sigma_{1}=v\left(x_{0},\sigma_{0}\right)=s_{⋆}$, and $\sigma_{2},\sigma_{3},\ldots ,\sigma_{n-1}$ are generated from $x_{1},x_{2},\ldots ,x_{n-1}$ as in (42).

Let *k* divide *n* and consider the lossless compression of $x_{0}^{n-1}$ in blocks of length *k*, $x_{j}=x_{jk+1}^{jk+k}$, j=0,1,…,n/k−1, by using the Shannon code, whose length function for a vector $x^{k}$ is $\left\lceil -\log Q_{\theta }\left(x^{k}\right)\right\rceil$. This is equivalent to predictive coding, where the prediction error signal, $z_{n}=x_{n}-f\left(x_{n-1},s_{n-1}\right)$ is compressed losslessly under a model of a memoryless source with a marginal $Q_{\theta }\left(z\right)$ (see Figure 2 for illustration). In this case, since the ceiling operation is carried over *k*-blocks, and there are n/k such *k*-blocks, the upper bound to $L(x^{n})$ becomes

(48) $$L\left(x^{n}\right)=\sum_{i=0}^{n/k-1}\left\lceil -\log Q_{\theta }\left(x_{ik+1}^{ik+k}\right)\right\rceil \leq \frac{1}{\theta }\cdot \sum_{i=1}^{n}\rho \left(x_{i}-u\left(x_{i-1},\sigma_{i-1}\right)\right)+n\log Z\left(\theta \right)+\frac{n}{k}.$$

On the other hand, the corresponding encoder of Figure 2 can be viewed as an encoder with $q\cdot M_{k}$ states, where $M_{k}=\left(\alpha^{k}-1\right)/\left(\alpha -1\right)$, since this is the number of combinations of a state of the *q*-state predictor and a state of the lossless block encoder, whose number of states is $\sum_{j=0}^{k-1}\alpha^{j}=M_{k}$. Thus,

(49) $$\frac{L(x^{n})}{n}\geq \hat{H}\left(X^{\ell }|X^{\ell -1}\right)-\frac{2\log\left(qM_{k}\right)+\left(qM_{k}-1\right)L_{\max}}{\ell }-\frac{L_{\max}}{n}$$

where it should be kept in mind that $L_{\max}$ is expected to grow linearly with *k*. Thus, by comparing the upper bound and the lower bound to $L(x^{n})$, we have

(50) $$\begin{array}{ccc} & & \frac{1}{n\theta }\cdot \sum_{i=1}^{n}\rho \left(x_{i}-u\left(x_{i-1},\sigma_{i-1}\right)\right)+\log Z\left(\theta \right)+\frac{1}{k}\\ & \geq & \hat{H}\left(X^{\ell }|X^{\ell -1}\right)-\frac{2\log\left(qM_{k}\right)+\left(qM_{k}-1\right)L_{\max}}{\ell }-\frac{L_{\max}}{n}. \end{array}$$

or, equivalently,

(51) $$\begin{array}{ccc} & & \frac{1}{n}\sum_{i=1}^{n}\rho \left(x_{i}-u\left(x_{i-1},\sigma_{i-1}\right)\right)\\ & \geq & \theta \left[\hat{H}\left(X^{\ell }|X^{\ell -1}\right)-\frac{2\log\left(qM_{k}\right)+\left(qM_{k}-1\right)L_{\max}}{\ell }-\frac{L_{\max}}{n}-\frac{1}{k}-\log Z\left(\theta \right)\right]. \end{array}$$

Maximizing the r.h.s. over θ≥0, we get

(52) $$\frac{1}{n}\sum_{i=1}^{n}\rho \left(x_{i+1}-u\left(x_{i},\sigma_{i}\right)\right)\geq \Delta \left(\hat{H}\left(X_{\ell }|X^{\ell -1}\right)-\frac{2\log\left(qM_{k}\right)+\left(qM_{k}-1\right)L_{\max}}{\ell }-\frac{L_{\max}}{n}-\frac{1}{k}\right).$$

The bound is meaningful if k≫1 and $\ell \gg qM_{k}$, so that the two subtracted terms in the argument of the function Δ(·) are small compared to the main term, $\hat{H}\left(X_{\ell }|X^{\ell -1}\right)$. It is tight essentially for sequences of the form $x_{i}=u\left(x_{i-1},\sigma_{i-1}\right)+z_{i}$, i=1,2,…, where $z^{n}=\left(z_{1},\ldots ,z_{n}\right)$ is typical to an i.i.d. source and where the marginal empirical distribution of each $z_{i}$ is close to $e^{-\rho (z)/\theta }/Z\left(\theta \right)$ for some θ≥0.

## 6. GKI in the Presence of Side Information

We will now discuss briefly an extension of the GKI for IL FS encoders in the case where SI is available at both the encoder and the decoder. The resulting condition is expressed in terms of the *joint spectral radius* (JSR) of a finite set of nonnegative matrices indexed by the various side-information symbols. We identify verifiable sufficient conditions for subexponential growth of Kraft sums and discuss the limitations inherent in the presence of SI.

Let X be the source alphabet as before and let W denote the finite alphabet of the SI sequence, $w_{1},w_{2},\ldots$, whose symbols are synchronized with the corresponding source symbols. As before, let Z be the finite set of states with |Z|=s. An FS encoder with SI is specified by an output function f:Z×X×W→Y, (Y being defined as a subset of ${\left\{0,1\right\}}^{*}$, similarly as before) and a next-state function g:Z×X×W→Z. Given an initial state, $z_{1}=z$, a source sequence, $x=(x_{1},x_{2},\ldots )$, and a SI sequence, $w=(w_{1},w_{2},\ldots )$, the encoder implements the equations:

(53) $$\begin{array}{ccc} y_{i} & = & f(z_{i},x_{i},w_{i}),\\ z_{i+1} & = & g(z_{i},x_{i},w_{i}), \end{array}$$

for i=1,2,…, and the total code-length produced by the encoder after *n* steps is

(54) $$L\left[f\left(z,x^{n},w^{n}\right)\right]=\sum_{i=1}^{n}L\left(f(z_{i},x_{i},w_{i})\right).$$

**Definition** **2.** *An FS encoder is said to be information-lossless with side information if for every n, the quadruple $\left(z_{1},y^{n},w^{n},z_{n+1}\right)\in Z\times Y^{n}\times W^{n}\times Z$ dictates $x^{n}\in X^{n}$.*

For each SI symbol, w∈W, define the corresponding Kraft matrix

(55) $${\left[K\left(w\right)\right]}_{zz^{\prime }}=\sum_{\left\{x\in X:\;g\left(z,x,w\right)=z^{\prime }\right\}}2^{-L[f(z,x,w)]},\;z,z^{\prime }\in Z.$$

Each K(w) is a nonnegative s×s matrix. For a given SI sequence, $w^{n}$, define the product matrix

(56) $$K\left(w^{n}\right)=K\left(w_{1}\right)\cdot K\left(w_{2}\right)\cdots K\left(w_{n}\right).$$

Now, let K={K(w),w∈W}. The growth rate of the Kraft products, $K(w^{n})$, over arbitrary SI sequences, $\left\{w^{n}\right\}$, is governed by the JSR of K, which is defined as follows.

**Definition** **3.** *The JSR of K is defined as*

(57) $$\rho_{\mathrm{JSR}}\left(K\right)=\lim_{n\to \infty }\max_{w^{n}\in W^{n}}{∥K\left(w^{n}\right)∥}^{1/n},$$

*where ∥·∥ is any matrix norm.*

It is a classical result that this limit exists and is independent of the chosen norm. The GKI in the presence of SI can be formulated as follows.

**Theorem** **3.** *For an IL FS encoder with SI,*

(58) $$\rho_{\mathrm{JSR}}\left(K\right)\leq 1.$$

**Proof.**  Fix an arbitrary SI sequence, $w^{n}\in W^{n}$ and states $z,z^{\prime }\in Z$. The $\left(z,z^{\prime }\right)$ entry of $K(w^{n})$ is given by

(59) $$\sum_{\left\{x^{n}:\;g\left(z,x^{n},w^{n}\right)=z^{\prime }\right\}}2^{-L[f\left(z,x^{n},w^{n}\right)]}.$$

Since the encoder is IL for the fixed sequence $w^{n}$, the mapping between $x^{n}$ and $\left(z,x^{n},w^{n},z^{\prime }\right)$ is injective over all paths from *z* to $z^{\prime }$. Grouping sequences according to their total code-length (similarly as before) and using a standard counting argument yield a linear upper bound (in *n*) on each matrix entry of $K(w^{n})$, uniformly over $w^{n}$. Exponential growth of $∥K(w^{n})∥$ is therefore impossible, and the JSR must satisfy $\rho_{\mathrm{JSR}}\left(K\right)\leq 1$. □

The following proposition can sometimes help. For example, if v=1 satisfies Proposition 1, this means that the Kraft sum is less than or equal to unity for every initial state and every SI sequence. In such a case, one can simply design a separate prefix code for every combination of initial state and SI sequence.

**Proposition** **1.** *If there exists a vector $v\in R^{s}$ with strictly positive components such that K(w)v≤v for every w∈W, then for every SI sequence $w^{n}$, $K(w^{n})v\leq v$, and hence the family $\left\{K\left(w^{n}\right)\right\}$ is uniformly bounded.*

**Proof.**  The claim follows by induction on *n*. Since v>0, uniform boundedness of all products implies $\rho_{\mathrm{JSR}}\left(K\right)\leq 1$. □

In contrast to the case without SI, bounding the spectral radius of each individual Kraft matrix K(w) is necessary but insufficient to control the growth rate of arbitrary products. In other words, even if ρ[K(w)]≤1 for every w∈W individually, the JSR may exceed unity, and in fact, may be arbitrarily large. As an example, let ϵ be an arbitrarily small positive real and consider the matrices

$$A=\left(\begin{array}{cc} \epsilon & \frac{1}{\epsilon }\\ 0 & \epsilon \end{array}\right)$$

and $B=A^{T}$. While ρ(A)=ρ(B)=ϵ, which is arbitrarily small, it turns out that

(60) $$\rho \left(A\cdot B\right)=\epsilon^{2}+\frac{1}{2\epsilon^{2}}+\sqrt{1+\frac{1}{4\epsilon^{4}}}\approx \frac{1}{\epsilon^{2}},$$

which is accordingly, arbitrarily large. The JSR is therefore the correct quantity governing feasibility.

Exact computation of the JSR is undecidable in general, even for nonnegative rational matrices. Consequently, the above result should be interpreted as a structural constraint rather than a computational criterion. Nonetheless, there is a plethora of upper and lower bounds to the JSR. Also, as mentioned earlier, the existence of a common positive sub-invariant vector provides a meaningful and verifiable sufficient condition for subexponential growth.

## 7. GKI for Lossy Compression

For lossy compression, we adopt a simple encoder model, where each source vector $x^{\ell }\in X^{\ell }$ is first mapped into a reproduction vector ${\hat{x}}^{\ell }=Q\left(x^{\ell }\right)\in {\hat{X}}^{\ell }$ within distortion ℓD and then ${\hat{x}}^{\ell }$ is losslessly compressed by an IL FS encoder with *s* states exactly as before. The latter may work in the level of single letters or in the level of *ℓ*-blocks. Let us define $B\left({\hat{x}}^{\ell }\right)=\left\{x^{\ell }\in X^{\ell }:\;d\left(x^{\ell },{\hat{x}}^{\ell }\right)\leq \ell D\right\}$ and let $B_{\ell }=\max_{{\hat{x}}^{\ell }\in {\hat{X}}^{\ell }}\left|B\left({\hat{x}}^{\ell }\right)\right|$. Now,

(61) $$\begin{array}{ccc} K_{zz^{\prime }} & \overset{▵}{=} & \sum_{\left\{x^{\ell }:\;g\left(z,Q\left(x^{\ell }\right)\right)=z^{\prime }\right\}}2^{-L[f\left(z,Q\left(x^{\ell }\right)\right)]}\\ & = & \sum_{\left\{{\hat{x}}^{\ell }:\;g\left(z,{\hat{x}}^{\ell }\right)\right)=z^{\prime }\}}\sum_{\left\{x^{\ell }:\;Q\left(x^{\ell }\right)={\hat{x}}^{\ell }\right\}}2^{-L[f\left(z,{\hat{x}}^{\ell }\right)]}\\ & \leq & \sum_{\left\{{\hat{x}}^{\ell }:\;g\left(z,{\hat{x}}^{\ell }\right)\right)=z^{\prime }\}}\left|B\left({\hat{x}}^{\ell }\right)\right|\cdot 2^{-L[f\left(z,{\hat{x}}^{\ell }\right)]}\\ & \leq & B_{\ell }\cdot \sum_{\left\{{\hat{x}}^{\ell }:\;g\left(z,{\hat{x}}^{\ell }\right)\right)=z^{\prime }\}}2^{-L[f\left(z,{\hat{x}}^{\ell }\right)]}\\ & \overset{▵}{=} & B_{\ell }\cdot {\hat{K}}_{zz^{\prime }}, \end{array}$$

and so, $K\leq B_{\ell }\cdot \hat{K}$ entry-wise. Now, $\hat{K}$ has all the properties that we have proved for the lossless case, it is just defined in the super-alphabet of *ℓ*-blocks. Since $\rho (\hat{K})\leq 1$, we readily have:

(62) $$\rho \left(K\right)=\rho \left(B_{\ell }\cdot \hat{K}\right)=B_{\ell }\cdot \rho \left(\hat{K}\right)\leq B_{\ell }.$$

Inequality (62) can be viewed as the FS analog of very similar earlier results derived in [7,8,9], for lossy *D*-semifaithful codes combined with UD codes, i.e., codes that consist of a cascade of a reproduction encoder (within distortion *D* as above) followed by UD lossless compression of the resulting reproduction vector. In those earlier articles, the main result was a generalized Kraft inequality, where the Kraft sum (or integral, in the continuous case) is upper bounded by the volume of a ball of normalized radius *D* in terms of the distortion measure, which, in essence, is exactly $B_{\ell }$.

For additive distortion measures, the quantity $B_{\ell }$ can be estimated using the method of types [15], or the Chernoff bound, or saddle-point integration [16,17]. If the method of types is used, then $B_{\ell }$ is upper bounded by $2^{\ell \Phi (D)}$, where

(63) $$\Phi \left(D\right)=\max_{\left\{P_{X\hat{X}}:\;d\left(X,\hat{X}\right)\leq D\right\}}H\left(X|\hat{X}\right).$$

Thus, the corresponding GKI reads

(64) $$\rho \left(K\right)\leq 2^{\ell \Phi (D)}.$$

But this bound is tight only in terms of the exponential order as a function of *ℓ* and hence is meaningful mainly for very large *ℓ*. For example, if the source and the reproduction vectors are binary, and the Hamming distortion measure is adopted, then it turns out that

(65) $$\Phi \left(D\right)=h_{2}\left(D\right)=-D\log_{2}D-\left(1-D\right)\log_{2}\left(1-D\right).$$

But we can say somewhat more in this case: here, $B_{\ell }$ is simply the cardinality of a Hamming sphere of radius ℓD, which upon careful analysis (see for example [17]), can be shown to be

(66) $$B_{\ell }=\frac{2^{\ell h_{2}\left(D\right)}}{\sqrt{2\pi \ell D(1-D)}}\cdot \left(1+o\left(1\right)\right).$$

In [9], more general results are available, including the case of multiple simultaneous distortion constraints. 

## Data Availability

No new data were created or analyzed in this study.
